# Epidemic Preparedness—*Leishmania tarentolae* as an Easy-to-Handle Tool to Produce Antigens for Viral Diagnosis: Application to COVID-19

**DOI:** 10.3389/fmicb.2021.736530

**Published:** 2021-12-13

**Authors:** Ilaria Varotto-Boccazzi, Alessandro Manenti, Francesca Dapporto, Louise J. Gourlay, Beatrice Bisaglia, Paolo Gabrieli, Federico Forneris, Silvia Faravelli, Valentina Bollati, Diego Rubolini, Gianvincenzo Zuccotti, Emanuele Montomoli, Sara Epis, Claudio Bandi

**Affiliations:** ^1^Department of Biosciences, University of Milan, Milan, Italy; ^2^Department of Biomedical and Clinical Science “L. Sacco”, University of Milan, Milan, Italy; ^3^VisMederi Research, Siena, Italy; ^4^The Armenise-Harvard Laboratory of Structural Biology, Department of Biology and Biotechnology “L. Spallanzani”, University of Pavia, Pavia, Italy; ^5^Department of Clinical Sciences and Community Health, University of Milan, Milan, Italy; ^6^Department of Environmental Science and Policy, University of Milan, Milan, Italy; ^7^Water Research Institute—National Research Council of Italy, IRSA−CNR, Brugherio, Italy; ^8^Pediatric CRC “Romeo ed Enrica Invernizzi”, University of Milan, Milan, Italy; ^9^Department of Molecular and Developmental Medicine, University of Siena, Siena, Italy

**Keywords:** serodiagnostics, *Leishmania tarentolae* expression system, epidemics, cell-factory, SARS-CoV-2, protein antigens

## Abstract

To detect and prevent emerging epidemics, discovery platforms are urgently needed, for the rapid development of diagnostic assays. Molecular diagnostic tests for COVID-19 were developed shortly after the isolation of SARS-CoV-2. However, serological tests based on antiviral antibody detection, revealing previous exposure to the virus, required longer testing phases, due to the need to obtain correctly folded and glycosylated antigens. The delay between the identification of a new virus and the development of reliable serodiagnostic tools limits our readiness to tackle future epidemics. We suggest that the protozoan *Leishmania tarentolae* can be used as an easy-to-handle microfactory for the rapid production of viral antigens to face emerging epidemics. We engineered *L. tarentolae* to express the SARS-CoV-2 receptor-binding domain (RBD) and we recorded the ability of the purified RBD antigen to detect SARS-CoV-2 infection in human sera, with a sensitivity and reproducibility comparable to that of a reference antigen produced in human cells. This is the first application of an antigen produced in *L. tarentolae* for the serodiagnosis of a Coronaviridae infection. On the basis of our results, we propose *L. tarentolae* as an effective system for viral antigen production, even in countries that lack high-technology cell factories.

## Introduction

The abrupt emergence of the COVID-19 pandemic has underlined the urgent need for new discovery platforms for the rapid development of diagnostic and monitoring tools, targeting infectious diseases (Ashour et al., 2020; Merad and Vabret, 2021). Indeed, the effective containment of an infectious disease depends on the immediate availability and application of diagnostic tools to detect infected individuals, as soon as possible after the first confirmed cases have emerged (World Health Organization, 2021). In fact, within a few weeks after the isolation of the virus responsible for COVID-19 (now called SARS-CoV-2), the viral genome had been sequenced (Shu and McCauley, 2017; Lu et al., 2020; Wu et al., 2020), and reverse transcription-quantitative PCR (RT-qPCR) diagnostic tools had been developed, for the detection of viral RNA (Islam and Iqbal, 2020). In contrast, the development of tools for the serological diagnosis of COVID-19, used to search for antibodies against the virus in patient blood or serum, required more time, owing to the need to produce the protein antigens to be implemented in the diagnostic tests (Long et al., 2020; Van Elslande et al., 2020). Protein antigens for serological diagnosis are generally produced using recombinant DNA technology, after the engineering or transfection of a cellular expression system, for the production of the desired antigen (Higgins and Hames, 1999; Yin et al., 2007; Puetz and Wurm, 2019; Tripathi and Shrivastava, 2019). The most widely used systems for the production of recombinant protein antigens include prokaryotes (e.g., *Escherichia coli*; Rosano and Ceccarelli, 2014), yeasts (e.g., *Saccharomyces cerevisiae*; Mattanovich et al., 2012), insect cells (through the baculovirus system; Bernard et al., 2001), and mammalian cells e.g., the human embryonic kidney cell line (HEK293, Khan, 2013). All these approaches have been employed for the production of different antigens from SARS-CoV-2 (Fujita et al., 2020; Tai et al., 2020; Walls et al., 2020; Wrapp et al., 2020; Djukic et al., 2021) and diagnostic kits, some of which are commercially available (e.g., see Milani et al., 2020). However, there is a general consensus that antigens produced in mammalian cells guarantee optimal performances in the diagnosis of human viral diseases (Sanchez-Garcia et al., 2016; Tripathi and Shrivastava, 2019). Indeed, the most widely used assays for the serological diagnosis of COVID-19 are based on antigens produced in human cells (e.g., the HEK293 cells). The differences in the diagnostic performances of viral antigens produced in mammalian cells, compared with those produced with other expression systems, mostly derive from the glycosylation pattern of the proteins and their folding (Sanchez-Garcia et al., 2016). In this context, human cells transfected with viral DNA are expected to produce protein antigens that are highly similar (or identical) to those produced by a human virus during its natural infection cycle. Diversely, prokaryotic systems, such as *E. coli*, are expected to produce non-glycosylated proteins, which would then potentially result in altered patterns in antibody detection, when used in the serological diagnosis of viral diseases. The limitations in the diagnostic performances of viral antigens produced in microbial prokaryotes are unfortunate because expression systems such as *E. coli* are easily engineered, cultured in optimized conditions in bioreactors, and very effective for high yield protein production at low cost (Yin et al., 2007; Rosano and Ceccarelli, 2014).

An alternative microbial system for recombinant protein production is *Leishmania tarentolae*, a eukaryotic microbe that infects reptiles, but is non-pathogenic to humans and other mammals. *L. tarentolae* is classified as a biosafety level class I organism and has already been developed as an expression system for recombinant mammalian proteins, due to the protein glycosylation pattern guaranteed by this microbe, which mimics that of vertebrates (Niimi, 2012; Legastelois et al., 2017; Klatt et al., 2019). Consequently, *L. tarentolae* is a very attractive system for antigen production, for both vaccine and diagnostic applications. To date, however, the use of this protozoon for the production of antigens for serodiagnosis has been very limited and has mainly been focused on antigens from protozoans of the genera *Leishmania* and *Trypanosoma*, with a sole application in the area of virology, on the hepatitis E virus-HEV (Baechlein et al., 2013; Rooney et al., 2015; De Souza et al., 2019; Rezaei et al., 2019; Murphy et al., 2020; Pereira et al., 2021).

Here, we show that *L. tarentolae* can easily be manipulated for the expression of a protein antigen from SARS-CoV-2 and that this antigen, tested against human sera, guarantees a diagnostic performance comparable with the same antigen produced in human cells. In the context of the recommendations to be prepared to face and combat emerging infections and future pandemics (World Health Organization, 2021), our study provides a proof of principle for the potential utility of the *L. tarentolae* expression system, as an easy-to-handle tool to rapidly respond to future epidemics, to produce viral antigens and for the accelerated development of serology-based diagnostic assays and population monitoring.

## Materials and Methods

### Plasmid Construction and *Leishmania tarentolae* Transfection

The sequence of SARS-CoV-2 Spike receptor-binding domain (RBD-SD1) was derived from the genomic sequence of the isolated virus “Severe acute respiratory syndrome coronavirus 2 Wuhan-Hi-1” released in January 2020, number MN908947 and comprises 819 nucleotides (range: 22,517–23,335) and 273 amino acids. The gene coding for RBD-SD1 was codon-optimized for *L. tarentolae*, synthesized and subcloned into the pLEXSY-sat2 vector (Jena Bioscience) for constitutive, secreted expression. Cloning results in the incorporation of a C-terminal 6xHis-tag onto the resulting Spike fragment, which comprises the RBD and SD1 domains (hereafter, Lt-RBD; Supplementary Text). The pLEXSY-sat2 vector integrates into the chromosomal 18S ribosomal RNA (ssu) locus of the *L. tarentolae* parasite. In addition, a signal sequence from *Leishmania mexicana*, which allows the secretion of the target protein into the culture medium, was added. The plasmid was cloned and propagated in *E. coli*, and then, the plasmid was linearized through digestion with SwaI enzyme. The host *L. tarentolae*–P10 was then transfected with the linearized plasmid by electroporation, according to the procedures of the manufacturer. Engineered strains were cultured in Brain Heart Infusion (BHI) liquid medium supplemented with porcine hemin (5 μg/ml, Jena Bioscience), penicillin–streptomycin (Pen-Strep, Jena Bioscience), and Nourseothricin (NTC, Jena Bioscience) (100 μg/ml) at 26°C in the dark under aerated conditions. For strain maintenance, cultures were diluted into fresh BHI medium twice a week.

### Evaluation of Receptor-Binding Domain-SD1 Expression

Expression of the target protein was evaluated by analyzing a sample of the supernatant from 10 recombinant clones of Lt-RBD by Western blotting. After 72 h of growth in BHI complete medium, supplemented with NTC at 26°C, *Leishmania* cultures were centrifuged 10 min at 3,000 g. Clarified supernatants were filtered using a 0.22-μm nitrocellulose membrane and concentrated at 5,000 g for 30 min, using an Amicon ultracentrifugal filter with a molecular weight (MW) cutoff of 10 kDa. Samples were diluted in a loading buffer 4X (Thermo Fisher Scientific), boiled for 5 min, and subsequently loaded onto a 4–20% gradient polyacrylamide gel (Bio-Rad Laboratories). After electrophoresis, proteins were transferred onto a nitrocellulose membrane (Bio-Rad Laboratories), according to the standard protocols, before blocking for 5 min at room temperature with EveryBlot Blocking Buffer (Bio-Rad Laboratories) and incubation with a 1:3,000 dilution of anti–6xHis-tag–horseradish peroxidase (HRP) antibody (Thermo Fisher Scientific) in the EveryBlot Blocking Buffer for 1 h. After three washes with phosphate-buffered saline (PBS) + 0.1% (v/v) Tween 20 (PBS-T), the membrane was incubated for 5 min with the Clarity Western ECL Substrate (Bio-Rad Laboratories) and detected using the ChemiDoc Touch Imaging System (Bio-Rad Laboratories).

### Large-Scale Expression and Purification of Lt-Receptor-Binding Domain

Recombinant strains expressing Lt-RBD were cultured for 4 days in complete BHI supplemented with NTC (100 μg/ml), Pen-Strep, and hemin (1.25 μg/ml) at 26°C in the dark and in constant agitation under aerated conditions. The *Leishmania* cultures (2.5 L) were centrifuged for 10 min at 3,000 g, and the supernatant was filtered on a 47-mm-diameter activated carbon filter (Merck) (1 filter/0.5 L) to help remove the hemin from the growth media, before a second filtration step on a 0.22-μm nitrocellulose membrane (Merck). The supernatant was concentrated to approximately 100 ml using the Vivaflow 200 system (Sartorius) and compatible PES Vivaflow 200 cassette (10,000 MW cutoff), at 4°C, before dialysis, using a dialysis membrane with a MW cutoff of 14–16 K (SpectaPor). Dialysis was carried out at 4°C in 2.6-L Binding Buffer [20 mM sodium phosphate buffer pH 7.4, containing 0.5 M NaCl and 0.01% (v/v) Tween 20]. The dialysate was transferred into fresh buffer (lacking Tween 20) after 4 h and then, after a third exchange, overnight at 4°C. Lt-RBD was purified on a 5-ml HisTrap Excel column (Cytiva), pre-equilibrated with Binding Buffer. The supernatant was loaded at a flow rate of 1 ml/min using AKTA basic fast protein liquid chromatography (FPLC) (Cytiva) and washed with Binding Buffer unit until the A280 nm reached baseline. Elution was carried out using a slow, stepwise (1, 3, 5, 10, 15, 20, 30, 50 70, and 100%) imidazole gradient, generated by mixing Binding Buffer with Elution Buffer (20 mM sodium phosphate buffer pH 7.4, 0.5 M NaCl, and 0.5 M imidazole), eluting at each step with four column volumes. After sodium dodecyl sulfate-polyacrylamide gel electrophoresis (SDS-PAGE) analysis, fractions containing the purified protein were pooled and concentrated to 500 μl, using an Amicon Ultra Filter unit with a MW cutoff of 10,000 (Millipore), and further purified by size exclusion chromatography on a Superdex 200 Increase 10/300 GL (Cytiva) column, pre-equilibrated in 1 × PBS (Merck) at a flow rate of 0.5 ml/min at room temperature. Peak fractions (1 ml per fraction) were pooled and aliquoted for conservation at −80°C. Protein concentration was measured spectrophotometrically using a NanoDrop, using the theoretically calculated A280 nm for 1 mg/ml Lt-RBD of 1.077 and with the BCA Protein Assay Kit (Merck).

### SDS-PAGE and Western Blot Analyses

The expression of purified Lt-RBD was evaluated both by Coomassie staining and Western blotting, as described above. In particular, the proteins were transferred to nitrocellulose membranes (Bio-Rad Laboratories) that were incubated for 5 min at room temperature with EveryBlot Blocking Buffer (Bio-Rad Laboratories) and then incubated with a 1:3,000 dilution of anti-SARS/SARS-CoV-2 Coronavirus Spike Protein (subunit 1) antibody (Thermo Fisher Scientific) in the blocking buffer for 1 h. After three washes with PBS-T, the membrane was incubated with a HRP-conjugated immunoglobulin G (IgG) anti-rabbit 1:30,000 (Thermo Fisher Scientific) in the blocking buffer for 1 h. Finally, after three washes with PBS-T, the membrane was incubated for 5 min with the Clarity Western ECL Substrate (Bio-Rad Laboratories) and detected by ChemiDoc Touch Imaging System (Bio-Rad Laboratories).

### Receptor-Binding Domain Production in Human (HEK293-F) Cells

For comparative analyses of glycosylation and MW determination (see below), we recombinantly produced RBD in human cells (hu-RBD), to simulate the viral protein produced during a natural infection in humans. The pCAGGS plasmid for production of the C-terminal His-tagged SARS-CoV-2 Spike RBD (#NR_52310) was obtained from BEI Resources (NY, United States). Recombinant SARS-CoV-2 Spike RBD was produced using HEK293-F cells (Invitrogen) cultivated in suspension using FreeStyle medium (Invitrogen) as described in Faravelli et al. (2021). The cell medium containing secreted SARS-Cov2 Spike RBD was collected 6 days after transfection. The sample was loaded onto a 5-ml His-Trap excel column (Cytiva) using a peristaltic pump and then eluted with a 0–250 mM imidazole gradient using a NGC FPLC system (Bio-Rad Laboratories). The eluted samples were subjected to immediate concentration to 1 mg ml^–1^ with concomitant buffer exchange with fresh PBS to remove imidazole using Amicon centrifugal filters (Merck), flash-frozen in liquid nitrogen, and kept at -80°C until usage. Before analysis, the protein samples were thawed and subjected to gel filtration using a Superdex 200 10/300 increase column (Cytiva) equilibrated with 25 mM HEPES/NaOH, 150 mM NaCl, pH 7.2 (Bruni et al., 2020).

### Size Exclusion Chromatography–Multiangle Light Scattering Analysis

Recombinant SARS-CoV-2 RBDs hu-RBD and Lt-RBD (20 μl of 1 mg/ml) were injected into a Protein KW-802.5 analytical size-exclusion column (Shodex) and separated with a flow rate of 1 ml min^–1^ in PBS using a high-pressure liquid chromatography system (Shimadzu Prominence). For MW characterization, light scattering was measured with a miniDAWN MALS detector (Wyatt), connected to a differential refractive index detector (Shimadzu RID-20A) for quantitation of the total mass, and to a UV detector (Shimadzu SPD-20A), for evaluation of the sole protein content. Chromatograms were collected and analyzed using the glycoconjugate analysis algorithm available in the ASTRA7 software (Wyatt, using an estimated dn/dc value of 0.185 ml/g for proteins and 0.140 ml/g for glycans). Calibration of the instrument was verified by injection of 10 μl of monomeric bovine serum albumin (3 mg/L) (Sigma-Aldrich).

### Differential Scanning Fluorimetry

DSF assays on recombinant SARS-CoV-2 Spike RBD samples (hu-RBD and Lt-RBD) at a concentration of 1 mg/ml in PBS buffer were performed using a Tycho NT.6 instrument (NanoTemper Technologies GmbH). Data were analyzed and plotted using the GraphPad Prism 7 (Graphpad Software).

### Serum Samples

For the setup and standardization of the in-house ELISA test (see below), we relied on the human sera reported in Table 1. Specifically, we used a commercially available IgG- and IgM-positive human serum sample from a convalescent COVID-19 subject (BIOIVT cod. 368424) as a positive control, a depleted human serum lacking IgA/IgG/IgM (Sigma-Aldrich), a negative human serum provided by the University of Milan (UNICORN study; Milani et al., 2020), and a pool of heterologous sera [HCoV positive serum BIOIVT cod. 406910-SR1; Pertussis Antiserum 1st IS-WHO international standard; human Influenza antibody to A/California/7/2009 “like” (H1N1v) virus (2nd international standard); Diphtheria antitoxin human IgG (1st international standard)].

**TABLE 1:** Serum samples and controls used to evaluate specificity parameters.

Positive control (HP-HS)	Human serum COVID-19 IgG/IgM BIOIVT cod. 368424-SR1
Negative control (NS-HS)	Negative human serum, Minus IgA/IgM/IgG
Pool of heterologous sera (HP-HET)	BIOIVT cod. 406910-SR1; Pertussis Antiserum (human) 1st IS-WHO international Standard; Influenza antibody (human) to A/California/7/2009 “like” (H1N1v) virus (2nd International Standard); Diphtheria antitoxin human IgG (1st International Standard).
Pos-Het (HP-HS/HP-HET)	Positive antibody response for homologous virus mixed with heterologous serum (ratio 1:1)
Pos-Neg (HP-HS/NS-HS)	Positive antibody response for homologous virus mixed with negative serum sample (ratio 1:1)
Negative human serum sample	Negative human serum sample provided by the University of Milan (UNICORN study) (Milani et al., 2020)

For the titration of IgG-specific antibodies, we relied on 10 commercially available human sera, both from symptomatic and asymptomatic donors, positive for the SARS-CoV-2 antibody response (CUST-BB-19032021-1A and CUST-BB-19032021-1B-NeoBiotech) and five pre-pandemic sera (year 2015) kindly provided by the University of Siena as negative controls. We further performed a comparative analysis to assess the validity of the in-house Lt-RBD ELISA on a cohort of 80 human sera collected from asymptomatic subjects in March/April 2020, provided by the University of Milan (UNICORN study; Milani et al., 2020). These sera were from (i) asymptomatic subjects that tested IgG seropositive for SARS-CoV-2 at the ELISA assay and (ii) asymptomatic subjects that tested seronegative and had no history of SARS-CoV-2 infection; all of these subjects tested PCR negative for the virus (Milani et al., 2020). ELISA assays on the above two groups of sera were performed using both a commercial RBD (see below) and the in-house Lt-RBD antigen.

### In-House *Leishmania*-Receptor-Binding Domain ELISA IgG

The IgG ELISA assay was qualified for the serological detection of SARS-CoV-2–specific antibodies in human serum samples using the Lt-RBD antigen according to International Conference on Harmonization Guidelines on Validation of Analytical Procedures [Q2 (R1)] (ICH, 1995).

Before starting the ELISA assay qualification experiments, a series of preliminary assays were performed to select the optimal antigen concentration. To this aim, coating concentrations of 1, 2, 3, and 4 μg/ml were tested against human positive and negative sera for SARS-CoV-2 and compared with Spike-RBD (1 μg/ml; commercially available from Sino Biological, China; hereafter, com-RBD) expressed and purified from HEK293 cells.

The setup and qualification experiments, as well as antibody titration and the comparative analysis, were performed as described below. ELISA plates were coated with purified recombinant Lt-RBD (2 μg/ml). After overnight incubation at 4°C, coated plates were washed three times with ELISA washing solution (300 μl per well) containing tris-buffered saline (TBS)–0.05% (v/v) Tween 20 (TBS-T) and then blocked for 1 h at 37°C with a solution of TBS containing 5% (w/v) non-fat dry milk (NFDM; Euroclone, Pero, Italy). Human serum samples were heat-inactivated at 56°C for 1 h to reduce the risk of the presence of live virus in the sample. Subsequently, twofold serial dilutions, starting from 1:100 in TBS-T containing 5% NFDM, were performed up to 1:51,200. Plates were washed three times, as previously; then, 100 μl of each serial dilution was added to the coated plates and incubated for 1 h at 37°C. Next, after the washing step, Goat anti-human IgG-Fc HRP-conjugated antibody (100 μl per well; 1:100,000; Bethyl Laboratories, Montgomery, United States) were added. Plates were incubated at 37°C for 30 min. After incubation, plates were washed, and 3,3’,5,5’-tetramethylbenzidine (100 μl per well) substrate (Bethyl Laboratories, Montgomery, United States) was added and incubated in the dark at room temperature for 20 min. The reaction was stopped by adding 100 μl of ELISA stop solution (Bethyl Laboratories, Montgomery, United States) and read within 20 min at optical density at 450 nm (OD_450_) using a SpectraMax ELISA plate (Medical Device) reader.

A cutoff value was obtained for each plate by multiplying by three the blank OD_450_ value and derived from six wells containing sample diluents and the secondary HRP antibody, but no serum. The performance of this cutoff calculation method was previously evaluated through a blinded study of asymptomatic subjects positive for SARS-CoV-2 (Milani et al., 2020). Samples whose OD_450_ was below the cutoff value at 1:100 dilution were scored as negative, whereas samples whose OD_450_ was above the cutoff value were scored as positive.

The IgG antibody titration was performed by a twofold serial dilution of human sera (from 1:100 to 1:25,600) using the above ELISA protocol and was expressed as arbitrary units. Samples for the comparative analysis on the 80 human sera were assayed with a 1:100 dilution and the OD_450_ value was used as a proxy of the IgG content. All readings were made in duplicate, and the mean value was used in the analyses.

### Qualification Criteria and Statistical Analyses

#### Specificity

All samples were tested with a starting dilution of 1:100 in four repetitions per plate and in two different plates, to generate four reportable (RP) values per sample. The percentage of geometric standard deviation (%GSD) was calculated between the RP values (RP) [GSD% = (eSD-1) × 100], where SD is the standard deviation between the natural logarithm (ln) results of the RP values.

#### Precision

Precision of the assay was assessed by testing positive control as neat and pre-diluted 1:2, 1:4, 1:8, 1:16, 1:32, 1:64, and 1:128 in dilution buffer; thus, considering the sample in first well is always diluted (1:100), the effective samples starting dilution in the first well will be 1:100 1:200, 1:400, 1:800, 1:1,600, 1:3,200, 1:6,400, and 1:12,800. The above mentioned sample dilutions will be tested eight times in two different plates from one operator on day 1 (four repetitions per plate); the RP value will be calculated between two determination results from the two different plates. The same testing activity will be performed by another operator on day 2 to obtain a total of eight RP values for each dilution (four RP values from operator 1 and four RP values from operator 2).

##### Precision—Linearity

Linearity was evaluated by performing a linear regression analysis of the base-2 logarithm serum dilution against the base-2 logarithm of the geometric mean titer (GMT) of all the eight RP values of the precision experiments using the method of least squares. The coefficient of determination, y-intercept, and slope of the regression line were calculated and reported.

#### Accuracy

The accuracy of the test was evaluated using the RP values obtained for the evaluation of the precision. According to International Conference on Harmonisation (ICH) guideline Q2 (ICH, 1995), the accuracy can be tested using either a conventional true value or an accepted reference value. The true value was calculated from the linearity result as GMT between the eight RP values: the GMT result of RP values from the neat sample were divided for the respective dilution factor to be investigated and compared with the real obtained value during testing (as GMT between the obtained RP values). Relative accuracy was evaluated by calculating the percentage of recovery on the GMT of the RP and the expected (true) titer obtained using this formula: 100*(GMT observed/GMT expected).

#### Robustness

Plates are incubated for 30 min (standard condition) with detection antibody to detect IgG specifically; to assess the influence of this incubation time, plates were incubated with the dilution antibody for 30 min at 37°C and two other different times: 20 and 40 min.

The positive and negative controls were tested with a starting dilution of 1:100 in four repetitions, in two plates in two different days for each condition to obtain four RP values.

## Results

### Lt-Receptor-Binding Domain-SD1 Protein Sequence Analysis, Expression, and Purification

**FIGURE 1:**
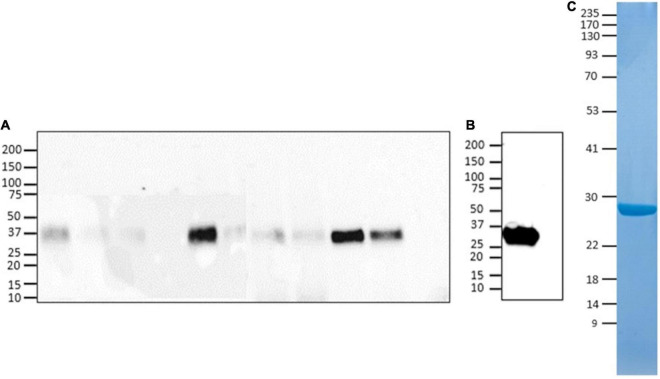
Evaluation of Lt-RBD protein production in *L. tarentolae*. **(A)** Expression analyses of Lt-RBD in the concentrated supernatant of 10 engineered *L. tarentolae* clones. A band of approximately 35 kDa is visible using an anti–His-tag antibody. **(B,C)** Analysis of Lt-RBD protein expression, purified by affinity chromatography, and confirmed by Western Blotting using an anti–SARS/SARS-CoV-2 Coronavirus Spike Protein antibody **(B)** and by SDS-PAGE with Coomassie staining **(C)**.

For expression of recombinant Lt-RBD protein, residues 22517–23335 of the SARS-CoV-2 genome (GenBank, MN908947) were cloned into the pLEXSY-sat2 vector, as described in the “Materials and Methods” section. The selected sequence encodes for the RBD domain (273 amino acids) of the SARS-CoV-2 Spike protein and also includes the SD1 subdomain of subunit S1. The inclusion of the SD1 portion was based on the evidence that this fragment is highly immunogenic (Wrapp et al., 2020). Lt-RBD was successfully expressed as a secreted protein into the *L. tarentolae* culture medium, as confirmed by SDS-PAGE and Western blotting using an anti–His-tag antibody (section “Materials and Methods”). The protein migrated by SDS-PAGE as a single band of approximately 35 kDa, corresponding to its estimated MW; this band was visible for most of the tested clones (Figure 1A). The supernatant from the non-engineered *L. tarentolae*–P10 parasite was used as a negative control. The most productive clone was selected for large-scale (2.5 L) expression and purification, via affinity chromatography and size exclusion chromatography (section “Materials and Methods”). Large-scale expression of Lt-RBD resulted in a yield of approximately 2.3 mg/L. The protein was identified by Western blotting using a specific anti-RBD antibody (Figure 1B) and judged to be pure by Coomassie blue staining (Figure 1C).

### Size Exclusion Chromatography–Multiangle Light Scattering and Differential Scanning Fluorimetry Analyses

**FIGURE 2:**
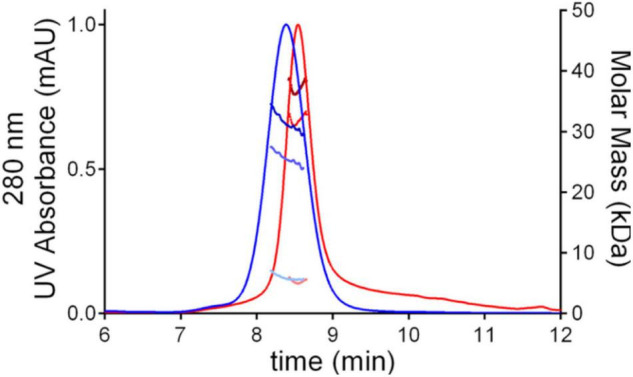
SEC-MALS chromatograms of SARS-CoV-2 RBD produced in HEK293F cells, hu-RBD (blue), and in *L. tarentolae*, Lt-RBD (red). For each sample, the molar mass values associated with the glycosylated RBD molecules, the protein-only fraction, and the glycan fraction of each peak are shown using dark, intermediate, and light dots, respectively.

SEC-MALS analysis showed that the purified protein is monodisperse with a total MW of 36.5 kDa, which is 5 kDa greater than the MW predicted on the basis of amino acid composition, due to the glycan moieties (Figure 2 and Table 2). Molecular masses determined for hu-RBD and Lt-RBD were 31.3 and 36.5 kDa, respectively.

**TABLE 2:** Summary of SEC-MALS analysis.

	Hu-RBD	Lt-RBD
Input values for analysis		
dn/dc (glycoprotein) (mg/L)*^a^*	0.185	0.185
dn/dc (glycan) (mg/L)*^b^*	0.140	0.140
280 nm Extinction coefficient (M^–1^ cm^–1^)*^c^*	1.3	1.39
Predicted molecular weight (protein only) (g/mol)*^c^*	25,921	31,547
Results from SEC-MALS analysis		
Total mass (g/mol)	31,320 ± 1,180	36,490 ± 1,540
Protein only (g/mol)	25,520 ± 960	31,560 ± 1,330
Glycan only (g/mol)	5,794 ± 1,195	4,935 ± 1,553

*^a^A standard dn/dc value for proteins of 0.185 mg/L was used as suggested by the MALS manufacturer.*

*^b^A standard dn/dc value for glycans of 0.140 mg/L was used as suggested by the MALS manufacturer.*

*^c^Extinction coefficients and molecular weights were estimated from protein sequences using the ExPASy ProtParam tool (Gasteiger et al., 2005; ExPASy, 2021). Mean values are reported with their associated standard deviation.*

**FIGURE 3:**
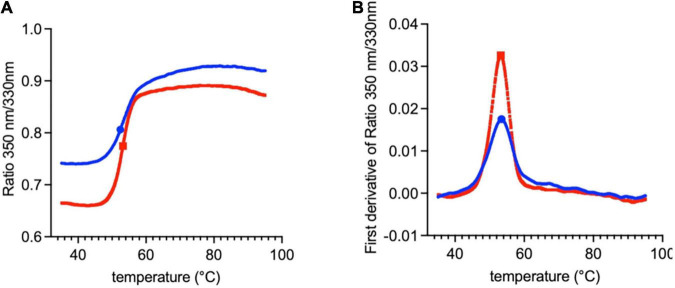
DSF traces of SARS-CoV-2 RBD produced in HEK293F cells (blue) and in *L. tarentolae* (red). The raw traces **(A)** and the first derivative **(B)** of the variation of the ratio of the protein intrinsic fluorescence at 350 and 330 nm during a DSF experiment are shown.

The difference between the molecular masses of the two proteins is mainly due to the inclusion of the SD1 subdomain in Lt-RBD. Furthermore, their unfolding temperatures, determined by DSF analysis, are highly similar (Lt-RBD-SD1: 53.2°C; hu-RBD: 53.4°C), indicating that Lt-RBD is as stable as the hu-RBD protein (Figure 3).

### Setup and Standardization of in-House ELISA

The RBD-ELISA qualification assay was carried out, as described by Mazzini et al. (2021). Purified recombinant Lt-RBD was tested for its ability to detect specific human antibodies. The protein was evaluated using four coating concentrations (1, 2, 3, and 4 μg/ml). The optimal concentration chosen for antigen coating was 2 μg/ml, and the optimal dilution for the secondary HRP conjugated anti-human IgG was 1:100,000 (Supplementary Table 1).

#### Specificity

The specificity was determined as the ability of the assay to differentiate between similar analytes and particularly to differentiate the target analyte from non-target analytes (World Organisation for Animal Health, OIE, 2013). To determine the specificity of the method for anti–SARS-CoV-2 IgG antibodies, positive samples for homologous and heterologous viruses/pathogens were tested. The ELISA specificity was evaluated testing the samples reported in Table 1.

The negative sample, as the heterologous sample, showed negative titers in all performed measurements of this testing series. The RP values for the sample mixtures Pos-Neg and Pos-Het samples showed a GSD% of 15.09%, which meets the acceptance criterion set at ≤ 50%. The positive homologous serum sample showed positive results across eight measurements in total. The negative human serum sample showed negative values, with the starting ODs (1:100 dilution) under the cutoff value established for this validation. Overall, the results reported in Supplementary Table 2 indicate that the assay is specific.

#### Precision

The precision of an analytical procedure is generally defined as the standard deviation or relative standard deviation (coefficient of variation) of a series of measurements (ICH, 1995; United States Pharmacopeia, USP, 2008). The standard deviation may be evaluated at three levels: repeatability, intermediate precision, and reproducibility.

##### Precision—Repeatability

Repeatability (also called intra-assay precision) shows the precision of the assay when the test is carried out in a laboratory over a relatively short time period using the same operator and equipment. Repeatability is assessed by evaluating variation of replicates (ICH, 1995; United States Pharmacopeia, USP, 2008; World Organisation for Animal Health, OIE, 2013).

The %GSD between four RP values from each operator (in one experiment on the same day) from the precision experiment was calculated and reported for each dilution, for each operator. The negative sample provided negative titer results. The positive sample showed GSD% of < 50% for all dilutions (Supplementary Table 3). These results indicate that the assay is repeatable for intra-assay precision parameters.

##### Precision—Intermediate Precision

The purpose of intermediate precision is to determine the capacity of the assay to provide reproducible results when random events occur. Variations could include operators, equipment, different days, and reagents. The intermediate precision was evaluated by performing two different assays by different operators using different sessions of analysis in the same laboratory. The %GSD between the GMT of the four RP values from each operator from the precision experiment was calculated and reported for each dilution. The positive sample showed GSD% of < 50% for all dilutions, which indicates that the assay meets the criteria for intermediate precision (Supplementary Table 4).

##### Precision—Linearity

The term “linearity” refers to the linearity of the relationship between the concentration and the assay measurement (United States Pharmacopeia, USP, 2008). This parameter needs to be demonstrated directly for the tested analyte and to be evaluated by visual examination of a plot of signals as a function of analyte concentration (ICH, 1995). The aim of linearity is to provide a model, linear or not, that is suitable to illustrate the relationship between concentration and response to the analyte (United States Pharmacopeia, USP, 2008). The classical acceptance criteria for linearity require that the correlation coefficient of the linear regression line is close to 1, the slope showing an absolute value between 0.7 and 1.3. In the case of a significant non-zero intercept, it is necessary to demonstrate that it does not have consequences on the accuracy of the assay (World Organisation for Animal Health, OIE, 2013). The correlation between sample dilutions and corresponding RP values, in the full dilutions range applied (1:100–1:12,800), was high, in that the coefficient of determination *R*^2^ was close to 1 (0.995), whereas the absolute value of the slope was 1.090 (Supplementary Table 5 and Supplementary Figure 1).

#### Accuracy

The assay quantitation range is the range within which the assay has demonstrated to have a suitable level of precision and accuracy. In detail, this range is defined by the lower and the upper sample dilutions able to provide linear and accurate results in agreement with the acceptability requirements. Although the last dilution points are almost all below the limit of detection, considering that the inter-assay and intra-assay precision are confirming the assay repeatability, the results of this validation process indicate that the assay is linear and precise along the whole range of dilutions applied in this validation resulting in a titer range between 71.0 and 14,061.8 (calculated as the GMT between the neat HP-HS results in precision experiments), corresponding to the closest dilutional point of 100 and 26,500, respectively (Supplementary Table 6). Although the last dilution point (1/128) is below the detection limit, the recovery value for each sample dilution was excellent.

#### Robustness

The robustness gives an indication of the assay reliability when events could occur during testing in a single laboratory. Plates are incubated for 20, 30, and 40 min to assess the influence of this incubation time with HRP. For the three different conditions of incubation time for antibody detection, the %GSD of the results for the positive control was 6.72%, which meets the acceptance criterion set at < 50%, and the negative control showed negative results; hence, the results indicate that the assay is robust (Supplementary Table 7). The qualification process of this specific IgG ELISA test for SARS-CoV-2, set up with the use of the Lt-RBD protein produced in *L. tarentolae*, fulfilled all the acceptability criteria. In particular, the assay demonstrated to be specific, reproducible, precise, linear, accurate, and robust. Thus, the procedure is defined as reliable and valid for the serological detection of SARS-CoV-2 IgG-specific antibodies.

### Detection of IgG Antibodies in Human Sera

**FIGURE 4:**
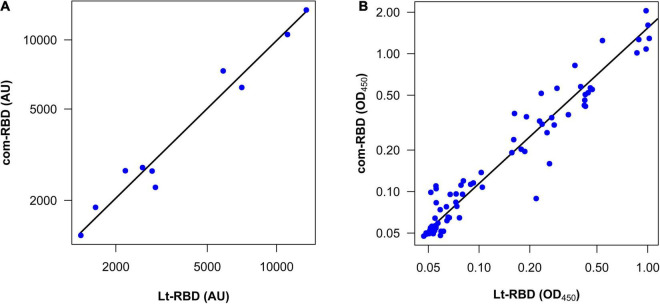
Correlation between the com-RBD and Lt-RBD antibody responses of human sera. **(A)** Antibody titers of 10 COVID-19–positive samples (*n* = 10); **(B)** OD_450_ values of COVID-19–positive and COVID-19–negative human sera (*n* = 80).

The ability of Lt-RBD to detect antibodies in comparison with the commercial RBD produced in HEK cells (com-RBD) was evaluated testing 15 human sera, among which ten positive for SARS-CoV-2. As negative controls, five pre-pandemic (2015) sera were included in the assay. The IgG antibody titers were calculated, and the results obtained with the two proteins were compared. As shown in Table 3, the results, expressed as antibody titers, were highly congruent. All five negative controls were classified as such when exposed to both the Lt-RBD and the com-RBD antigens according to the previously defined cutoff values (Table 3). When considering the 10 SARS-CoV-2 positive sera, there was a strong correlation between the IgG antibody titers obtained with the Lt-RBD and the com-RBD antigens [*r* = 0.98 (95% CI 0.95–0.99)]. Moreover, the slope of a major axis regression (Warton et al., 2006) of com-RBD on Lt-RBD antibody titers (log_10_-transformed values) was not significantly different from 1 [estimate = 0.98 (95% CI 0.84–1.15)], and the intercept was not significantly different from 0 [estimate = 0.07 (95% CI -0.49–0.63)], indicating a strictly direct proportionality between the antibody titers obtained with the two methods (Figure 4A).

**TABLE 3:** Antibody titers of 10 COVID-19–positive and five pre-pandemic (negative) human sera determined using Lt-RBD antigen produced in *L. tarentolae* and com-RBD antigen produced in HEK cells.

		IgG antibody titer (AU)	
Sample ID	Description	Lt-RBD-SD1	com-RBD
1	Positive	7,327.1	5,860.9
2	Positive	2,279.3	2,973.0
3	Positive	13,497.0	13,479.7
4	Positive	2,692.8	2,199.6
5	Positive	6,206.9	7,060.2
6	Positive	1,408.1	1,408.2
7	Positive	10,557.0	11,159.9
8	Positive	2,684.3	2,875.9
9	Positive	2,780.0	2,613.3
10	Positive	1,865.5	1,631.8
11	Negative	<100	<100
12	Negative	<100	<100
13	Negative	<100	<100
14	Negative	<100	<100
15	Negative	<100	<100

The comparative analysis performed on the 80 human sera showed that all samples, but one, were classified in a coherent way (positive or negative) according to the pre-defined cutoff values (Supplementary Table 8). The only mismatch that we obtained was from a serum sample whose OD_450_ was very close to the cutoff (Supplementary Table 8). From the quantitative viewpoint, there was a strong correlation between the Lt-RBD and com-RBD OD_450_ values of the same subjects [*r* = 0.94 (95% CI 0.92–0.96)]. However, the OD_450_ of Lt-RBD (median value = 0.075) was significantly lower than those of the com-RBD (median value = 0.097) (Wilcoxon matched-pairs rank test, V = 438, *P* < 0.001), and the slope of a major axis regression of com-RBD on Lt-RBD OD_450_ (log_10_-transformed values) was significantly larger than 1 [estimate = 1.13 (95% CI 1.06–1.20)]; the elevation was also significantly larger than 0 [estimate = 0.19 (95% CI 0.19–0.25)]. These statistics indicate that Lt-RBD provided slightly but consistently lower OD_450_ values compared to the com-RBD, possibly because of the slightly higher MW of the Lt-RBD-SD1 protein, which may affect the performance of the ELISA assay (Figure 4B).

## Discussion

Antigens produced in the *L. tarentolae* expression system have been assayed for the serological diagnosis of *Leishmania* infections in humans and dogs, with successful results both in terms of sensibility and sensitivity (De Souza et al., 2019; Rezaei et al., 2019; Pereira et al., 2021). In addition, in parasites from the *Trypanosoma* genus, which are phylogenetically related to *Leishmania* (within the family Trypanosomatidae), antigens produced in *L. tarentolae* showed satisfactory performances in serological assays, for both African and American trypanosomiases (Rooney et al., 2015; Murphy et al., 2020). These few studies, on the use of antigens produced in *L. tarentolae* for the serological diagnosis of *Leishmania* and *Trypanosoma* infections, have been largely based on the assumption that protein production in phylogenetically related organisms would ensure proper folding and glycosylation, thus better serodiagnostic performance, in comparison with antigens produced in distantly related organisms, such as yeast, and perhaps by a positive bias toward *L. tarentolae* in people working on pathogenic species of *Leishmania* and *Trypanosoma*. Indeed, to the best of our knowledge, no studies have thus far been published on the use of antigens produced in *L. tarentolae* for serodiagnosis of infections caused by bacteria or protozoa not belonging to the Trypanosomatidae. In addition, there is only one application to the serodiagnosis of a viral infection, caused by the hepatitis E virus (Baechlein et al., 2013).

The success in the serodiagnostic use of antigens produced in *L. tarentolae* to detect *Leishmania* spp. or *Trypanosoma* spp. infections does not guarantee the efficacy of this protein microfactory as a source of antigens for the diagnosis of infectious diseases caused by other pathogens. Using SARS-CoV-2 as a study system, our present work provides the first sound evidence on the potential utility of *L. tarentolae* as a microfactory to produce antigens for serodiagnosis of Coronaviridae infections. Our results are summarized as follows: (i) the model antigen that we selected for this study, i.e., the RBD protein fragment from SARS-CoV-2, was effectively produced and purified and demonstrated to possess folding and glycosylation patterns comparable to that of the same antigen produced in mammalian cells; (ii) all of the tests carried out to validate an in-house ELISA assay based on Lt-RBD met the established recommendation criteria; (iii) titrations on standard COVID-19–positive sera showed linearity in the recorded values, and a perfect match was observed in the attribution of the sera to the two reference classes (positive or negative), using Lt-RBD or com-RBD; (iv) finally, the analysis with Lt-RBD on 80 sera from a previous study led to an almost perfect match with the results obtained with com-RBD (with just one mismatch).

The gold standard for serological diagnosis viral infections in humans and other mammals is the use of antigens produced in mammalian cells, ideally in cells form the same species (Sanchez-Garcia et al., 2016; Tripathi and Shrivastava, 2019; Walls et al., 2020; Wrapp et al., 2020). However, the use of mammalian cells to produce viral antigens might present potential limitations, particularly for rapid application and fast production in developing countries: highly specialized cell factories are indeed needed for efficient protein production in mammalian cells; production costs are relatively high compared to microbial-based systems; there is a relatively high risk of viral infection and contamination of cultured cells (Hartmann and Breitling, 2014; Taheri et al., 2016; Klatt et al., 2019). In the case of *L. tarentolae*, the risk for a viral contamination of the cultures cannot be excluded, although there is currently no evidence for viral infections in this system, and the transmission of viruses from humans and animals to *Leishmania* cells can be regarded as highly improbable (Hartmann and Breitling, 2014). Certainly, in comparison with mammalian cells, *Leishmania* system produces moderate quantities of proteins (up to 30 mg/L; Basile and Peticca, 2009); in particular, in agreement with our results, reported productions of viral proteins in this system are in the range of 2–4 mg/L. Moreover, although most authors emphasize that glycosylation patterns in *L. tarentolae* and humans are very similar (e.g., Niimi, 2012; Legastelois et al., 2017; Klatt et al., 2019), minor differences in post-translation modifications (PTMs) of recombinant antigens might anyway alter their antibody binding capacity (Klausberger et al., 2021); in addition, PTMs could vary even within the same species, in relation with the life cycle stage and culture conditions (McConville et al., 2002; Klatt et al., 2013). N-glycosylation in *L. tarentolae* was investigated in recombinant human EPO and was demonstrated to be highly homogeneous, with a biantennary glycosylation profile that shares its core with that typical of mammals (Breitling et al., 2002). However, higher branched N-glycans and terminal sialic acid residues (e.g., N-Acetyl-Neuraminic acid) have not been documented in proteins expressed in *L. tarentolae* (Breitling et al., 2002; Klatt et al., 2019). In addition, current knowledge on O-glycosylation in *L. tarentolae* proteins is limited (Klatt et al., 2013). In summary, the presence of antigenically relevant differences in the PTMs of proteins produced in *Leishmania* and human cells cannot be excluded. However, our results indicate that the RBD antigen produced in *L. tarentolae* guarantees a reliable serodiagnosis of SARS-CoV-2 infection, in agreement with previous results obtained on HEV (Baechlein et al., 2013).

Finally, our study underlines *L. tarentolae* as a system that is easy to manipulate and culture, providing a sound alternative for the rapid production of serodiagnostic proteins, even at the forefront of novel epidemics, e.g., in the presence of “spillover” events in tropical countries (Olival et al., 2017). For example, the production of proteins by *Leishmania* cells can be obtained in flasks or simple bioreactors, within 2–4 days from the starting of the culture (Fritsche et al., 2007), whereas mammalian systems, such as the HEK cells, require longer times 4–6 days (Faravelli et al., 2021). We emphasize that protein production in *L. tarentolae* holds the potential to be scaled to the industrial level, growing the parasites in bioreactors and harvesting proteins using high throughput strategies (Niimi, 2012).

In addition, in comparison with yeast and insect cells, *L. tarentolae* is expected to ensure the production of viral antigens whose glycosylation pattern mimics that determined after expression in mammalian cells (Niimi, 2012; Legastelois et al., 2017; Klatt et al., 2019) while in the presence of few differences (see discussion above). In comparison with prokaryote-based expression systems, the advantages of *L. tarentolae* are the production of proteins with mammalian-like PTMs and the solubility of the proteins (Klatt et al., 2013). Of course, *L. tarentolae* presents possible disadvantages compared to prokaryotic expression systems: the engineering and the selection of transformed clones is more laborious; yield and production costs are not competitive with those ensured by well-established systems such as *E. coli* (Taheri et al., 2016).

## Data Availability Statement

The original contributions presented in the study are included in the article/Supplementary Material, further inquiries can be directed to the corresponding author.

## Ethics Statement

The studies involving human participants were reviewed and approved by the Ethics Committee of the University of Milan, approval number 17/20, approval date March 6, 2020. The patients/participants provided their written informed consent to participate in this study.

## Author Contributions

CB and SE conceived and coordinated the study. IV-B and BB contributed to cell culture and *Leishmania* maintenance. IV-B, FD, and VB performed the set-up and serological assay. AM contributed to the design of the serological assays and analyzed the data. DR performed the statistical analyses. LG, PG, FF, and SF performed the biochemical analyses. IV-B, FD, and AM wrote the first draft of the manuscript. GZ, EM, SE, and CB revised the manuscript. All authors have read and agreed to the published version of the manuscript.

## Conflict of Interest

The antigen Lt-RBD and its potential application have been described in the patent application no. IT 02021000004160; publication date 23 February 2021. AM, FD, and EM were employed by VisMederi Srl. The remaining authors declare that the research was conducted in the absence of any commercial or financial relationships that could be construed as a potential conflict of interest.

## Publisher’s Note

All claims expressed in this article are solely those of the authors and do not necessarily represent those of their affiliated organizations, or those of the publisher, the editors and the reviewers. Any product that may be evaluated in this article, or claim that may be made by its manufacturer, is not guaranteed or endorsed by the publisher.

## Supplementary Material

Supplementary Figure 1 Linearity graph showing the correlation between ELISA titer and serum dilution.
