# Supplementary figures and images for: Epidemic Preparedness—Leishmania tarentolae as an Easy-to-Handle Tool to Produce Antigens for Viral Diagnosis: Application to COVID-19
Source: Front Microbiol. 2021 Dec 13;12:736530. doi: 10.3389/fmicb.2021.736530 (PMC8710741; doi:10.3389/fmicb.2021.736530)

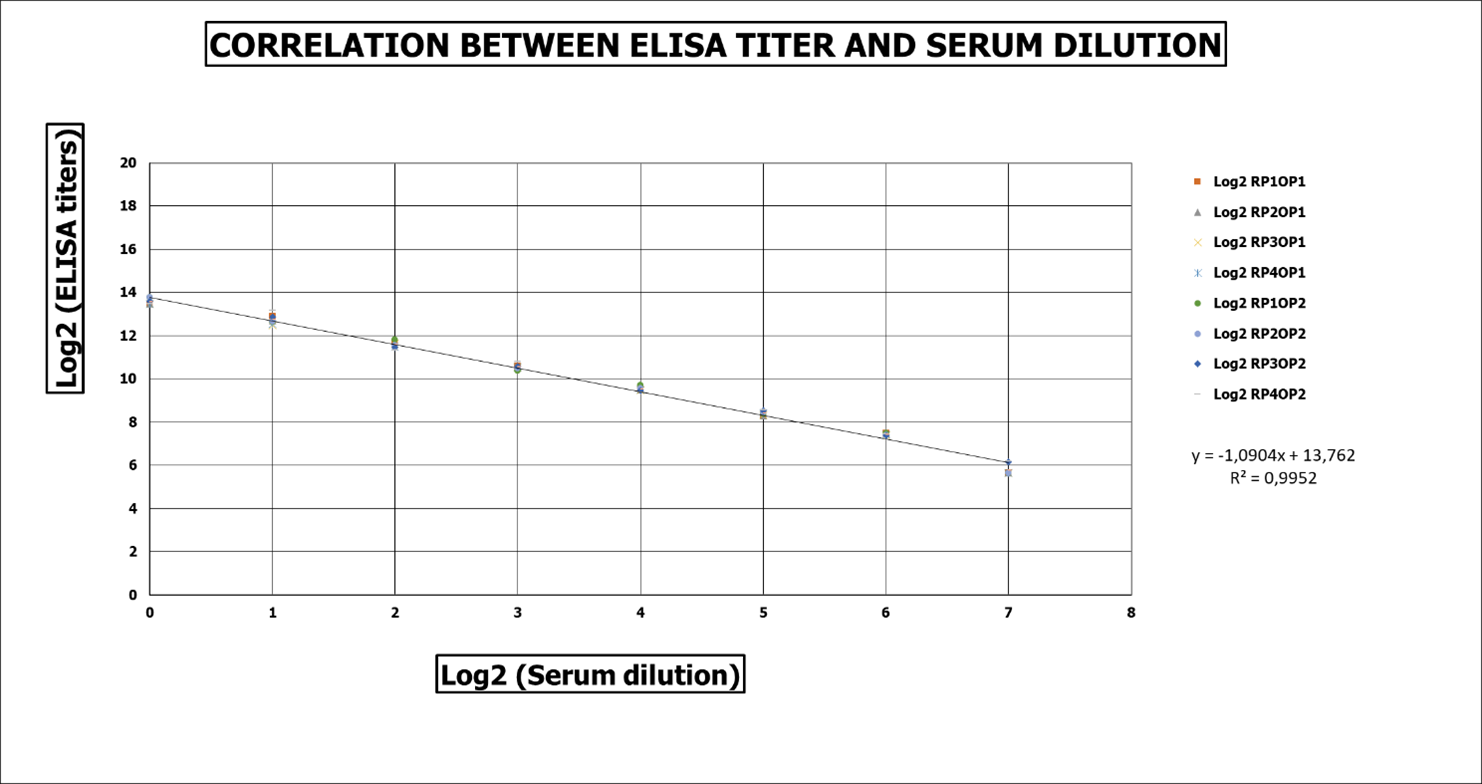

Supplement: Supplementary Figure 1 — Linearity graph showing the correlation between ELISA titer and serum dilution. [file Data_Sheet_1.zip › Supplementary Figure S1.TIF]
